# Cloning and Partial Characterization of an Endo-α-(1→6)-d-Mannanase Gene from *Bacillus circulans*

**DOI:** 10.3390/ijms20246244

**Published:** 2019-12-11

**Authors:** Shiva kumar Angala, Wei Li, Zuzana Palčeková, Lu Zou, Todd L. Lowary, Michael R. McNeil, Mary Jackson

**Affiliations:** 1 Mycobacteria Research Laboratories, Department of Microbiology, Immunology and Pathology, Colorado State University, Fort Collins, CO 80523-1682, USA; weili@colostate.edu (W.L.); Zuzana.Svetlikova@colostate.edu (Z.P.); M.Mcneil@ColoState.EDU (M.R.M.); 2 Department of Chemistry, The University of Alberta, Edmonton, AB T6G 2G2, Canada; Lu.Zou@gilead.com (L.Z.); tlowary@ualberta.ca (T.L.L.)

**Keywords:** endo-α-(1**→**6)-d-mannase, mannoside, *Mycobacterium*, lipomannan, lipoarabinomannan, phosphatidylinositol mannosides

## Abstract

Mycobacteria produce two major lipoglycans, lipomannan (LM) and lipoarabinomannan (LAM), whose broad array of biological activities are tightly related to the fine details of their structure. However, the heterogeneity of these molecules in terms of internal and terminal covalent modifications and complex internal branching patterns represent significant obstacles to their structural characterization. Previously, an endo-α-(1→6)-D-mannanase from *Bacillus circulans* proved useful in cleaving the mannan backbone of LM and LAM, allowing the reducing end of these molecules to be identified as Man*p*-(1→6) [Man*p*-(1→2)]-Ino. Although first reported 45 years ago, no easily accessible form of this enzyme was available to the research community, a fact that may in part be explained by a lack of knowledge of its complete gene sequence. Here, we report on the successful cloning of the complete endo-α-(1→6)-D-mannanase gene from *Bacillus circulans* TN-31, herein referred to as *emn*. We further report on the successful production and purification of the glycosyl hydrolase domain of this enzyme and its use to gain further insight into its substrate specificity using synthetic mannoside acceptors as well as LM and phosphatidyl-*myo*-inositol mannoside precursors purified from mycobacteria.

## 1. Introduction

Two complex α-(1→6)-linked D-mannose-containing lipoglycans, lipomannan (LM) and lipoarabinomannan (LAM) populate the cell envelope of mycobacteria [1]. They are essential to the integrity of the cell envelope [2] and play important roles in the immunopathogenesis of mycobacterial infections [1,3,4]. LM and LAM are believed to share a mannosylated phosphatidyl-*myo*-inositol lipid anchor and a mannan backbone composed of α-(1→6)-linked-D-mannopyranosyl residues occasionally branched with α-(1→2)-D-mannopyranosyl residues [1]. Despite recent advances in the biosynthetic pathway of these lipoglycans, the fine details of the structure of their mannan backbone, specifically, the precise distribution of the α-(1→2)-D-mannopyranosyl residues along the mannan backbone, is not known with certainty. To help in these analyses, two types of glycosyl hydrolases capable of hydrolyzing the mannan backbone of LM and LAM have traditionally been used: An exo-type α-(1→2,3,6)-mannosidase (EC # 3.2.1.24), which cleaves the terminal mannopyranosyl residues from the non-reducing end [5] and an endo-α-(1→6)-D-mannanase (EC # 3.2.1.101), which cleaves the glycosidic bond between two internal mannopyranosyl residues [6]. While the first enzyme can be purified from Jack bean and is commercially available, to the best of our knowledge, no commercial source of the second enzyme is available. The endomannanase hydrolyzing α-(1→6)-D-mannopyranosyl residues was discovered in 1974 by Ballou and co-workers [7] who then went on to purify this enzyme from the soil bacterium, *Bacillus circulans* [8], and subsequently shared this enzyme with other laboratories [6,9]. Some 24 years later, the gene encoding this enzyme, *aman6*, was identified by reverse genetics [10] but found to encode a protein only about half the size of that purified by Ballou et al., even though the recombinant product of the gene apparently displayed the expected catalytic activity on an (undefined) α-(1→6)-D-mannan substrate releasing 6α-mannotriose and 6α-mannobiose [10] [Figure S1]. Efforts by our laboratory to re-clone this gene by PCR based on its published sequence consistently failed, suggestive of potential rearrangements in the *Sau*3AI-digested chromosomal DNA libraries used in the isolation of *aman6* [10]. As a part of our continued effort to decipher the biosynthetic pathway of LM and LAM and establish the fine details of their structure, here, we report on the cloning of the full-size endo-α-(1→6)-D-mannanase gene (which we named *emn*) and its use to recombinantly express and purify the glycosyl hydrolase domain of this enzyme. The cleavage properties of the glycosyl hydrolase domain of Emn on synthetic mannoside acceptors, purified LM from *Mycobacterium smegmatis* and biosynthetic precursors, phosphatidyl-*myo*-inositol di- and hexa-mannosides, were investigated using mass spectrometry.

## 2. Results and Discussion

### 2.1. Cloning of the Full-Size Endo-α-(1→6)-d-Mannanase Gene from Bacillus circulans TN-31

Using reverse genetics, Maruyama et al. identified *aman6* as the gene encoding the endo-α-(1→6)-D-mannanase from *B. circulans* TN-31 by screening a *Sau*3A1-digested genomic DNA library of this bacterium. The *aman6* gene was reported to encode a 589-amino acid-long protein with a 36 amino-acid signal peptide (GenBank accession number AB024331) [10]. Attempts to PCR-amplify *aman6* in our laboratory using primers to the reported nucleotide sequence were unsuccessful. The nucleotide BLAST analysis of *aman6* yielded a gene, *gymC10_1685* from *Paenibacillus sp.* Y412MC10, with 88% DNA sequence identity to *aman6* on the first 1722-bp but a substantially larger coding sequence totaling 3249-bp instead of 1767 bp for *aman6*. Using primers specific to the 5′-end of *aman6* and to the downstream region of *gymC10_1685*, a 3522 bp-long PCR product was amplified from *B. circulans TN-31 genomic DNA*. The fragment encompasses a 3255 bp-long open reading frame encoding a 1084 amino acid protein with a 33 amino acid signal peptide (Figure S1). The molecular weight of the mature protein (131 kDa) matches that of the endo-α-(1→6)-D-mannanase purified by Ballou and collaborators from the same bacterium [8]. We named the full-size endomannanase gene *emn*. The mature Emn protein consists of a glycosyl hydrolase family 76 (GH-76) catalytic domain from amino acids 36 to 360 followed by three consecutive family six carbohydrate-binding modules from residues 392 to 522, 534 to 657 and 670 to 793, and a domain of unknown function (DUF4959) from residues 811 to 902. The GH-76 CaZy family of glycosyl hydrolases consists of α-(1→6)-mannanases (EC # 3.2.1.101) and α-glucosidases (EC # 3.2.1.20), whereas family six carbohydrate-binding modules are non-catalytic domains thought to facilitate the binding of the catalytic GH domain to its substrate(s).

### 2.2. Expression and Purification of the Glycosyl Hydrolase Domain of Emn

The production of the mature Emn protein from a pET14b expression plasmid in *E. coli* BL21 pLysS proved to be toxic to the cells and failed to yield any detectable protein product. We were, however, able to produce, using the same expression system, a 43 kDa N-terminally His_6_-tagged recombinant protein encompassing the glycosyl hydrolase domain of Emn (hereafter referred to as GH-emn) corresponding to residues 36 to 400 of the full-size enzyme (Figure S1) and to purify it to near-homogeneity using a combination of nickel affinity chromatography and gel filtration (Figure S2). The purified GH-emn protein was stored at –80 °C in 20 mM Tris pH 7.5 buffer containing 150 mM NaCl and 20% glycerol for over 24 months without any appreciable loss of activity.

### 2.3. Digestion of Synthetic Mannoside Substrates

**Figure 1:**
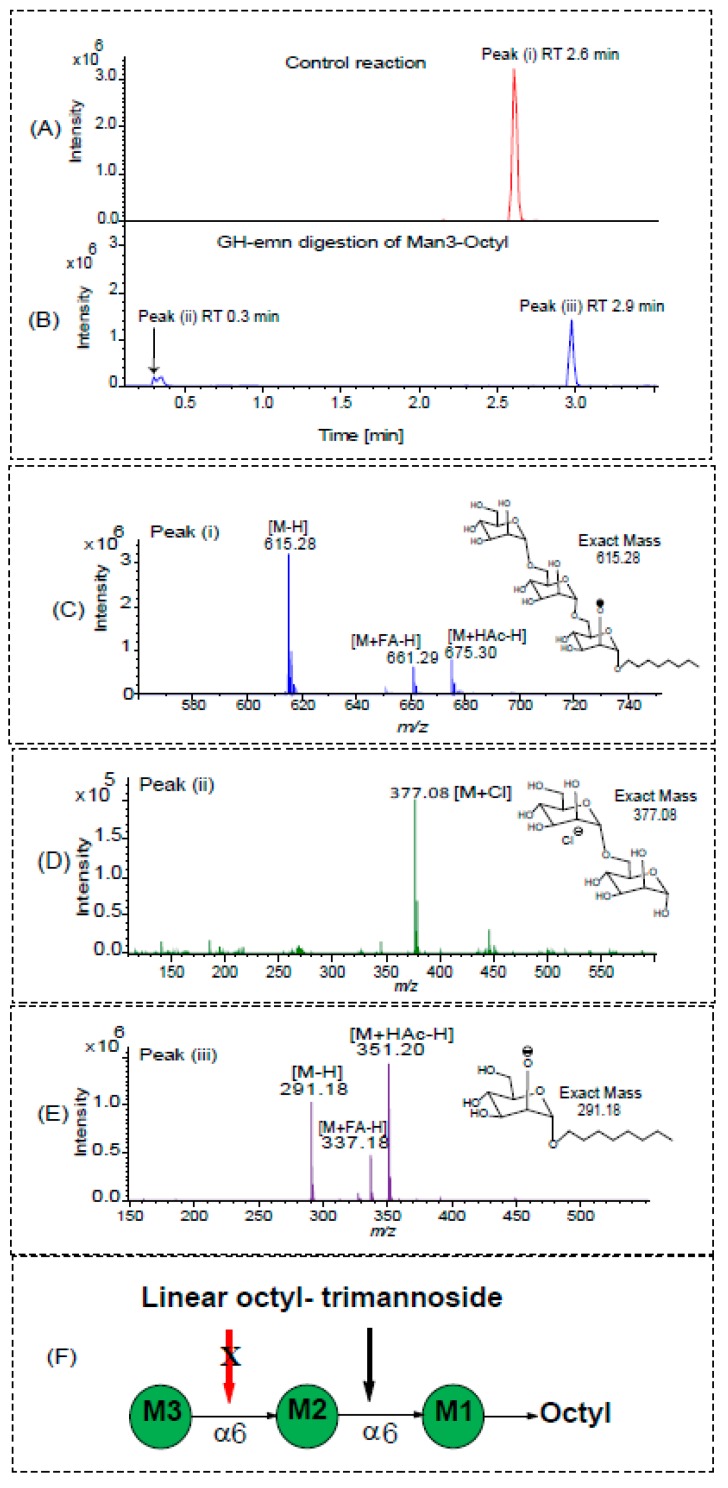
LC/MS analysis of the GH-emn-digested synthetic octyl trimannoside. (**A**,**B**) Base peak chromatograms showing the elution profiles of the substrate (**A**) and products of the enzymatic reaction (**B**). (**C**) Mass spectrum of the undigested synthetic octyl trimannoside at *m/z* 615.28 [M-H] as well as formate and acetate adducts. (**D**,**E**) Mass spectra of the products of the reaction: mannobiose (peak (ii)) (**D**) with a free reducing end at *m/z* 377.08 [M+Cl^–^] and Man*p*_1_-octyl (peak (iii)) (**E**) at *m/z* 291.18 [M-H], 337.18 (M+formate) and 351.2 (M+acetate). (**F**) Cartoon showing the location of the mannosyl cleavage (thicker arrow) of the octyl trimannoside at residue M2. The red arrow indicates the absence of cleavage by the enzyme.

The purified GH domain of Emn was then tested for glycosyl hydrolase activity using two synthetic unbranched linear substrates consisting of octyl trimannoside (*α-*d*-*Man*p*-(1→6)-α-d-Man-(1→6)-α-d-Man-(1→octyl)) and octyl pentamannoside (α-d-Man*p*-(1→6)-α-d-Man-(1→6)-α-d-Man-(1→6)-α-d-Man-(1→6)-α-d-Man-(1→octyl)). These substrates are structurally similar to those originally used by Ballou and co-workers [8] to study the substrate specificity of the purified, native, full-size enzyme. Those used by Ballou were unsubstituted at the reducing end, whereas those we evaluated harbor an octyl aglycon at their reducing end to facilitate the identification of the hydrolytic products of the reaction and thus, exactly where the enzyme cleaves. Enzyme assays were carried out as described under Materials and Methods in liquid chromatography—mass spectrometry (LC/MS) sample vials at 50 °C for 16 h [8]—after which the reaction mixture was directly injected into the LC/MS instrument without further purification. Figure 1A–B shows the base peak chromatograms of octyl trimannoside and its GH-emn digestion products. The mass spectrum of the only major peak found for the substrate (peak (i)) showed a major [M–H] ion at *m/z* 615.29 as well as an ion at *m/z* 675.30 [M+HAc–H] and an ion at *m/z* 661.29 [M+HCOOH–H], all corresponding to the parent molecule, Man*p*_3_-(1→octyl) (Figure 1C). In contrast, the base peak chromatogram of the enzymatic products revealed the loss of peak (i) with the concomitant appearance of two new peaks, (ii) and (iii). The mass spectrum of peak (ii) showed a strong ion at *m/z* 377.08 [M+Cl^–^] corresponding to Man*p*_2_ (Figure 1D), whereas peak (iii) yielded a signal at *m/z* 351.20 [M+HAc–H] corresponding to Man*p*-(1→octyl) (Figure 1E). These data show that the enzyme cleaves the trisaccharide at glycosidic bond of the middle Man*p* residue (labeled as M2 in Figure 1F) unit rather than the glycosidic bond of the Man*p* residue at either end of the trimannoside unit (labeled as M1 and M3 in Figure 1F).

**Figure 2:**
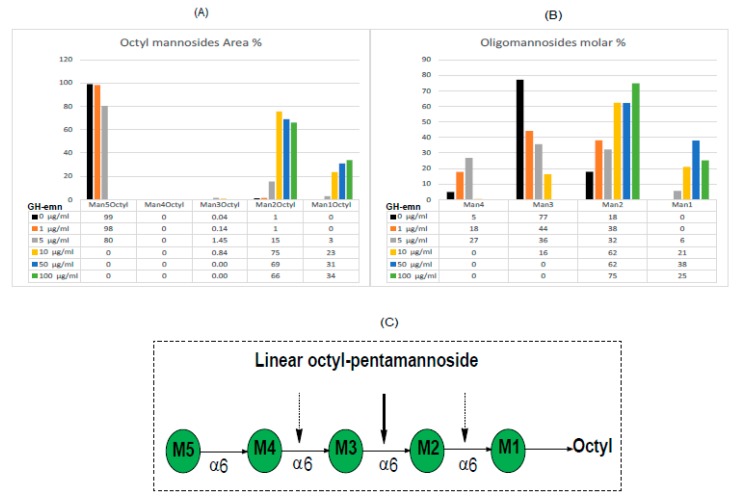
Digestion of octyl pentamannoside with increasing concentrations of GH-emn. (**A**) Percentage area of products containing an octyl chain. (**B**) Molar percentage of oligomannosides with a free reducing end. (**C**) Cartoon showing the locations of the three possible mannosyl cleavage sites. The thicker arrow denotes the dominant cleavage site having three mannosyl residues at the non-reducing end and the dotted arrows indicate other, less favored cleavage sites. The further cleavage of the released Man*p*_4_ and Man*p*_3_ is not shown.

A similar hydrolytic pattern was obtained upon digestion of the octyl pentamannoside by GH-emn (Figure 2). In general, Man*p*_1-4_ as free reducing components were found as [M+Cl^–^] ions and Man*p*_1, 2, 3,_ and _5_-(1→octyl) as [M+HAc–H] ions. The extracted ion chromatograms for these ions were integrated to obtain areas used to calculate the data presented in Figure 2A,B. The amounts of these ions varied with the enzyme concentration used (Figure 2A,B). Importantly, no Man*p*_4_-(1→octyl) was detected with any amount of enzyme, as predicted from the data of Man*p*_3_-(1→octyl) (Figure 1). Complete digestion occurred at the two highest enzyme concentrations (50 and 100 µg/mL) yielding only Man*p*_2_-(1→octyl) and Man*p*_1_-(1→octyl) in a ratio of approximately 2:1 and the free mannosides Man*p*_2_ and Man*p*_1_ in a 0.9:1 ratio. Traces of Man*p*_3_-(1→octyl) (Figure 2A) along with oligomannosides Man*p*_4_ and Man*p*_3_ (Figure 2B) were only detected when less enzyme was used in the assay, suggesting that these products are further cleaved to form Man*p*_1_-(1→octyl), and free Man*p*_2_ and Man*p*_1_. Collectively, these results indicate that GH-emn cleaves the octyl pentamannoside substrate at three different positions, as summarized in Figure 2C. At all three cleavage positions, the enzyme binds to three mannosyl residues and cuts between the middle Man and the reducing end Man. Consistently with that binding, the enzyme cannot further process Man*p*_2_-(1→octyl) or Man_2_, in agreement with previous studies from Dr. Ballou’s laboratory [8].

### 2.4. Digestion of Phosphatidyl-Myo-Inositol-Mannosides

The reducing end of LM and LAM consists of phosphatidyl-*myo*-inositol where the *myo*-inositol residue is mannosylated at positions C-2 and C-6. This lipid anchor may be esterified with up to four acyl chains [1]. Phosphatidyl-*myo*-inositol mannosides, also known as PIMs, may also exist as free glycolipids populating the inner and outer membranes of mycobacteria [11]. Tri- and tetra-acylated phosphatidyl-*myo*-inositol dimannosides (Ac_1_PIM_2_ and Ac_2_PIM_2_) and tri- and tetra-acylated phosphatidyl-*myo*-inositol hexamannosides (Ac_1_PIM_6_ and Ac_2_PIM_6_) are the most abundant form of PIMs found in the mycobacterial cell envelope [11]. As expected from the results above, GH-emn showed no activity on deacylated PIM_2_ (d-PIM_2_) which lacks α-(1→6)-linked mannosyl residues (Figure S3).

**Figure 3:**
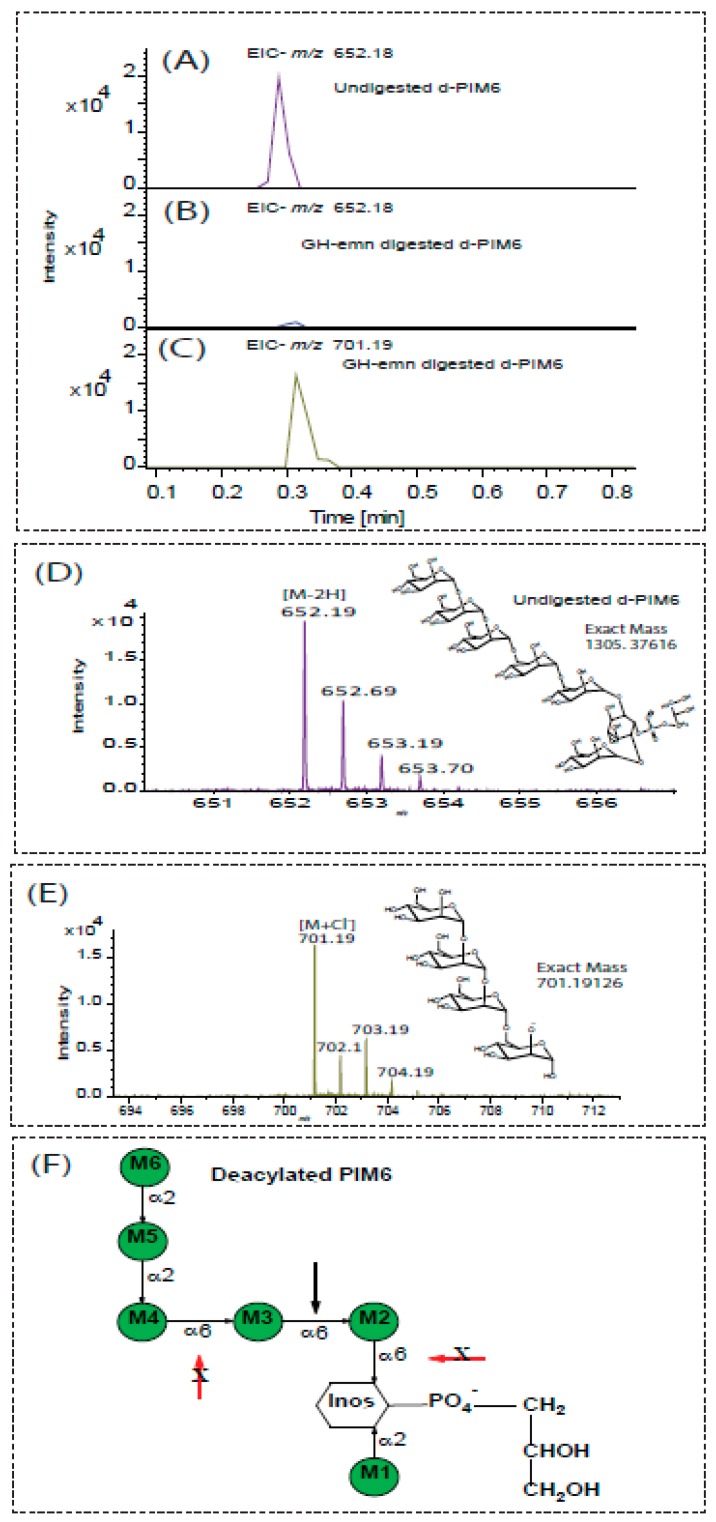
LC/MS analysis of undigested and GM-emn-digested deacylated PIM_6_. (**A**,**D**) Extracted Ion Chromatogram (**A**), structure (**D**), and mass spectrum (**D**) of d-PIM_6_ (*m/z* 652.18 [M–2H]) before enzyme treatment. (B) Extracted ion chromatograms (EIC) of d-PIM_6_ (*m/z* 652.18 [M–2H]) after digestion with GH-emn. The EIC of *m/z* 701.19 [M+Cl^–^] shown in panel (**C**) corresponds to the dominant tetramannoside product of d-PIM_6_ after digestion with GH-emn. Its mass spectrum is shown in panel (**E**). (**F**) Cartoon showing the location of the mannosyl cleavage site of GH-emn to form Man_4_. The red arrows indicate the absence of cleavage by the enzyme.

Since earlier work has established that purified, native, Emn lacks activity on yeast mannan in which the backbone is substituted with Man*p-*α-(1→3)- and α-(1→2)-linked residues [7,8], we sought to determine whether GH-emn could cleave the pentamannoside extending from the six-position of *myo*-inositol in deacylated Ac_2_PIM_6_ (d-PIM_6_). In this molecule, the first three mannosyl residues attached to *O*-6 of the inositol are linked via α-(1→6) glycosidic linkages and the last two mannosyl residues are present as the dimannoside α-Man*p*-(1→2)-α-Man*p* attached to position 2 of the third Man*p* from the inositol residue (Figure 3). PIM_6_, as received from BEI, is a mixture containing both PIM_2_ (shown above to not be a substrate for GH-emn) and PIM_6_ in equal amounts. Hence, we needed to focus our analysis on the released free mannosides rather than the inositol-containing components. GH-emn activity on intact d-PIM_6_ was analyzed by LC/MS in the negative ion mode. Upon enzymatic treatment, the amount of intact d-PIM_6_ (MS and structure shown in Figure 3D) decreased dramatically (Figure 3A,B). Concomitantly, the chromatogram and mass spectrum of the enzyme-treated sample were dominated by a peak at *m/z* 701.19 [M+Cl^–^] corresponding to the tetramannoside, D-Man*p*-(1→2)-α-D-Man*p*-(1→2)-α-D-Man*p*-(1→6)-α-D-Man*p*-(1→6) (Figure 3C,E). No ions corresponding to the mono-, di-, tri- or penta- mannosides were observed in the enzyme-treated sample. These results indicate that the GH-emn can tolerate an α-(1→2)-linked dimannoside substitution at the non-reducing terminus (M4 in Figure 3F) of the α-(1→6 trimannoside component (M4, M3, and M2) of d-PIM_6_.

### 2.5. Analysis of Endomannanase-Digested LAM and LM

**Figure 4:**
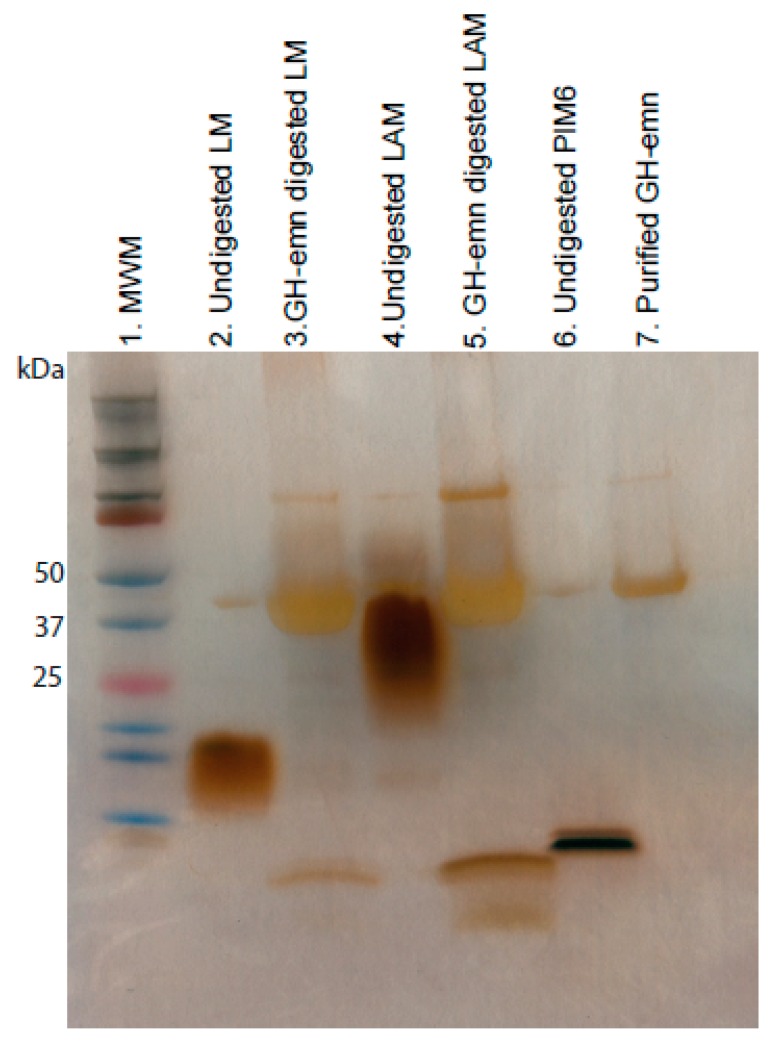
Silver-stained SDS PAGE showing the GH-emn digestion products of native *M. smegmatis* LM and LAM. MWM: Molecular weight marker.

Next, we tested the endomannanase activity of GM-emn on the acylated forms of LM and LAM from *M. smegmatis*. Complete digestion of these substrates after an overnight incubation with GH-emn was evident from the analysis of the products of the reaction by SDS-PAGE followed by silver staining (Figure 4). Enzymatic digestion of LM and LAM further resulted in a low molecular weight product migrating slightly faster than acylated PIM_6_ (Figure 4, lanes 3, 5 and 6), which we tentatively attribute to di- or tri-mannosylated forms of phosphatidyl-*myo*-inositol released from the reducing end of both lipoglycans.

**Figure 5:**
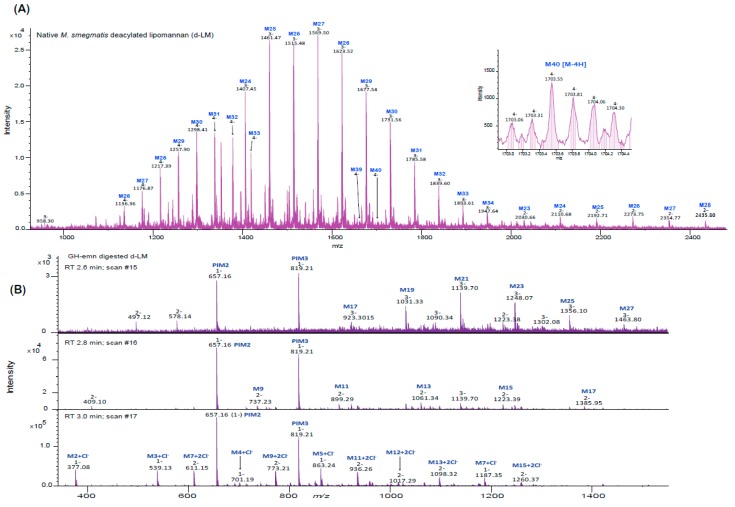
Negative ion mass spectra of deacylated LM from *M. smegmatis* before and after digestion with GH-emn. (**A**) Mass spectrum of undigested d-LM. The spectrum shows the mass distribution of the mannan backbone of d-LM which contains up to 40 mannosyl residues (differing by the mass of one hexose). Inset: The isotopic pattern for quadruply-charged d-LM with 40 mannosyl residues indicates the sensitivity of the mass spectrometer for high-molecular weight compounds. (**B**) Mass spectrum of d-LM after digestion by GH-emn. The mass spectra for three different retention times (RT) are shown. At all retention times, the spectra are dominated by singly-charged PIM_2_ and PIM_3_. RT 2.6 and 2.8 min show the presence of ions for Man17 to Man27 [M-3H] and Man9 to Man17 [M-2H]. RT 3.0 min shows low-molecular weight oligomannosides (Man_2_ to Man_7_) as singly charged and doubly charged chlorine adducts.

To gain further insight into the nature of the fragments released by GH-emn from deacylated *M. smegmatis* LM (d-LM), the products of the reaction were also analyzed by LC/MS. The LC/MS profile of undigested d-LM was dominated by doubly, triply and quadruply charged, long-chain, high-molecular weight (range of 2,700 to 6,800 Da) mannans containing 15 to 40 mannosyl residues (Figure 5A). The most abundant components appeared as triply charged series [M–3H] of ions at *m/z* 1406.77, 1460.79, 1514.81, 1568.82, 1622.84, 1676.86 and 1730.88 corresponding to LM molecules with 24 to 34 mannosyl residues. The less abundant quadruply-charged series [M–4H] corresponded to mannans with 27 to 40 mannosyl residues, while the least abundant doubly-charged series contained 24 to 33 mannosyl residues. Together, these results confirm that the mannan backbone of *M. smegmatis* LM is composed of up to 40 mannosyl residues. GH-emn digestion of this heterogeneous population of d-LM resulted in two major ions at *m/z* 657.16 and 819.21, corresponding to the mass of d-PIM_2_ and d-PIM_3_ released from the reducing end (Figure 5B). Additionally, the presence of oligomannans (with free reducing ends) was deduced from the presence of singly, doubly and triply charged series of ions matching the mass of Man*p*_2_ and higher oligomers with odd numbers of mannosyl residues from Man*p*_3_ to Man*p*_27_ (Figure 5B). These oligomannans result from the presence of *O*-2 mannosyl substitutions at various positions of the mannan backbone of LM, preventing GH-emn from cutting. Their detailed structure awaits further characterization.

## 3. Materials and Methods

### 3.1. Cloning, Expression and Purification the Glycosyl Hydrolase Domain of Emn

*B. circulans* TN-31 (ATCC^®^290101™) was obtained from the American Type Culture Collection and grown on agar medium at 30 °C as recommended by the ATCC. Genomic DNA was isolated using a standard extraction protocol. The coding sequence of the full-length mature protein (3150 bp) was amplified using primers Emn1 (5′-CTCGAGTATACCGCATCAGATGGGG-3′) and Emn2 (5′-CTCGAGTTACTCCAAGCCATCCTGCC-3′). The glycosyl hydrolase domain sequence of *emn* (GH-emn; 1092 bp) was PCR-amplified using primers GH-emn1 (5′-CATATGTATACCGCATCAGATGGGG-3′) and GH-emn2 (5′-CTCGAGTTAATTGTAGCGTTCCGCTTCGA-3′). Both PCR fragments were cloned into the *Nde*/ and *Xho*I sites of the pET14b expression plasmid and transformed into *E. coli* BL21 DE3 pLysS. For GH-emn production, freshly prepared LB broth containing 100 µg/mL ampicillin was inoculated with a preculture of pET14b-GH-emn transformant and incubated at 30 °C until optical density measured at 600 nm (OD_600_) reached 0.2. Gene expression was induced by adding isopropyl-β-thio-galactopyranoside (1 mM final concentration) to the culture medium. After 4 h of induction at 30 °C, cells were collected by centrifugation and resuspended in lysis buffer consisting of Tris-HCl pH 7.5 containing 150 mM NaCl, 1 mM MgCl_2_, 1 μg/mL DNase and protease inhibitor cocktail (Sigma, St. Louis, MO, USA). Cells were broken using a French press (1500 psi; 3 passages) and cell debris were removed by ultracentrifugation at 4 °C for 30 min at 110,000 × g. The supernatant was then applied to a His-trap column (GE Healthcare, Pittsburgh, PA, USA) and the protein eluted in a gradient of 10–500 mM imidazole, prior to gel filtration using a Superdex 200 column (GE Healthcare). Aliquots of the purified GH-emn protein were stored at –80 °C in 20 mM Tris pH 7.5 buffer containing 150 mM NaCl and 20% glycerol until further use. Protein concentrations were determined using the Pierce BCA protein assay kit as recommended by the manufacturer.

### 3.2. Assays Using Synthetic Mannosides Substrates

*α-*d*-*Man*p*-(1→6)-α-d-Man-(1→6)-α-d-Man-(1→octyl] (octyl trimannoside-) and α-d-Man*p*-(1→6)-α-d-Man-(1→6)-α-d-Man-(1→6)-α-d-Man-(1→6)-α-d-Man-(1→octyl (octyl pentamannoside) were chemically synthesized as described [12,13]. Endomannanase reaction mixtures contained the synthetic mannosides (100 µM) and purified GH-emn (0 to 500 µg/mL) in a total volume of 100 µL in 10 mM ammonium acetate buffer (pH 6). Control reactions contained buffer in place of the purified enzyme. Enzymatic reactions were carried out in LC/MS vials at 50 °C for 16 h at which point the enzyme was inactivated by heating at 65 °C for 30 min [8]. In the experiment where increasing concentrations of GH-emn were used, the reactions mixture were incubated at 50 °C for 2 h instead of 16 h.

### 3.3. Assays Using Purified Mycobacterial PIMs and Lipoglycans

Purified *M. tuberculosis* H37Rv phosphatidyl-*myo*-inositol hexamannosides (PIM_6_) and *M. smegmatis* LAM were received from BEI resources. LM was extracted and purified from *M. smegmatis* whole cells as described previously [14]. Purified PIM_6_ (20 µg) was deacylated using monomethylamine following an established procedure [15] and further digested with GH-emn (0.25 mg/mL) in 10 mM ammonium acetate buffer (pH 6) for 24 h at 50 °C. Native LM from *M. smegmatis* (50 µg) was deacylated with 0.2 N NaOH (200 µL) at 37 °C and then neutralized with 10% aqueous glacial acetic acid. Deacylated LM (d-LM) was separated from sodium salts using an Amicon ultra-0.5 mL centrifugal filter (3 kDa MWCO) and resuspended in 10 mM ammonium acetate buffer (pH 6) prior to digestion with GH-emn as described for d-PIM_6_. D-LM and d-PIM_6_ before and after enzymatic digestion were analyzed by LC/MS (see next section).

Native LAM (40 µg) and LM (40 µg) from *M. smegmatis* digested with GH-emn (50 µg) in 100 mM citrate phosphate buffer pH 6.6 for 16 h at 50 °C were also was analyzed by SDS-PAGE followed by silver staining alongside 10 µg of undigested starting material.

### 3.4. Analysis of Substrates and Reaction Products by Liquid Chromatography—Mass Spectrometry

Separation of the GH-emn-digested products was performed using a reverse-phase X-bridge C18 column (50 mm × 2.1 mm; 1.7 µM) on a Waters ACQUITY UPLC system. The mobile phases used were water (solvent A), acetonitrile (solvent B) and 0.5 M ammonium acetate (solvent C) under the following gradient conditions: 0–0.3 min (10% B), 0.3–3 min (70% B), 3–4.8 min (98% B), and 4.8–6.8 min (10% B) at a flow rate of 400 µL/min. A constant 2% solvent C was maintained throughout the LC run.

Mass spectrometry (MS) was performed using a Bruker maXis plus II high-resolution quadrupole time-of-flight (Q-TOF). The electrospray ionization (ESI) source settings were as follows: end plate offset voltage 500 V, capillary voltage 3500 V, nebulizer gas pressure 3.0 bar, dry gas flow rate 10 L/min. Two different tune parameters were used for analyzing native and GH-emn-digested products. For detecting low molecular compounds, the tune parameters were: funnel RF 300 Vpp, multipole RF 300 Vpp, ion energy 3.0 eV, low mass range for ion transmission *m/z* 100, collision energy 8.0 eV, collision RF 450 Vpp, pre-pulse storage 5.0 µs, ion cooler RF 800 Vpp and transfer time 80.0 µs. For detecting high molecular weight, d-LM tune parameters were: funnel RF 400 Vpp, multipole RF 400 Vpp, ion energy 3.0 eV, low mass range for ion transmission *m/z* 600, collision energy 8.0 eV, collision RF 2500 Vpp, pre-pulse storage 5.0 µs, ion cooler RF 800 Vpp and transfer time 140 µs. A data analysis was performed using Bruker compass data analysis 4.4 SR1.

## 4. Conclusions

The full-length endo-α-(1→6)-D-mannanase gene from *B. circulans* TN-31 was successfully cloned for the first time and its glycosyl hydrolase domain expressed and purified in active and stable form from *E. coli*. The analysis of the digestion pattern of synthetic mannosides, PIM and LM provided further insights into the substrate specificity of the endomannanase domain of the enzyme. The availability of this enzyme, together with a better understanding of its catalytic activity, should greatly facilitate the structural analysis of α-(1→6)-D-mannan-containing polysaccharides, including mycobacterial LM and LAM.

## Supplementary Materials

Supplementary materials can be found at https://www.mdpi.com/1422-0067/20/24/6244/s1.
