# Supplementary material for: Cloning and Partial Characterization of an Endo-α-(1→6)-d-Mannanase Gene from Bacillus circulans
Source: Int J Mol Sci. 2019 Dec 11;20(24):6244. doi: 10.3390/ijms20246244 (PMC6940960; doi:10.3390/ijms20246244)
Supplement: Supplementary file 1 [file ijms-20-06244-s001.pdf]

Figure S1

1 M S L R S G Q L F R F L A V P L A I A L M L G S M P G I G T S K A Y A Y T A S D
1 ATGTCACTAAGATCAGGTCAACTTTTCAGGTTTCTGGCGGTGCCTCTGGCTATCGCGCTCATGCTCGGTTCCATGCCGGGCATCGGAACGTCCAAAGCCTACGCCTATACCGCATCAGAT
41 G D T A M K A F N D T F W D P N A K M F W K D S K R E K H Q D F W V E A E L W E
121 GGGGATACAGCGATGAAAGCTTTTAACGATACATTTTGGGATCCGAACGCCAAGATGTTCTGGAAGGACTCGAAGCGCGAAAAGCATCAAGACTTCTGGGTGGAGGCTGAGCTGTGGGAA
81 L V M D A Y Q H T S D P A L K A E L K T Q I D D V Y D G T V A K Y G Q D W T N N
241 TTGGTCATGGATGCGTATCAGCATACATCCGATCCTGCTTTGAAAGCCGAGCTTAAAACGCAAATCGACGATGTATATGACGGCACCGTCGCCAAGTACGGGCAAGATTGGACGAATAAC
121 P F N D D I M W W A M G S A R A Y Q I T G N P R Y L E A A R D H F D F V Y D T Q
361 CCGTTCAATGACGATATTATGTGGTGGGCGATGGGCAGCGCCAGAGCCTATCAAATCACCGGAAACCCAAGATATTTGGAAGCCGCGAGGGATCATTTCGATTTTGTGTACGATACGCAG
161 W D E E F A N G G I W W L N S D H N T K N A C I N F P A A Q A A L Y L Y D I T K
481 TGGGATGAAGAGTTCGCAAACGGCGGCATTTGGTGGCTGAACAGCGACCATAATACCAAAAATGCGTGCATTAATTTCCCGGCGGCGCAAGCGGCGCTTTATCTTTACGATATTACGAAA
201 D E H Y L N A A T K I F R W G K T M L T D G N G K V F D R I E I E H G A V P D A
601 GATGAGCATTATCTAAACGCGGCAACCAAAATATTCAGATGGGGCAAAACGATGCTTACGGACGGAAACGGAAAAGTGTTCGACCGCATCGAAATTGAACATGGCGCGGTTCCGGATGCC
241 T H Y N Q G T Y I G S A V G L Y K A T G N A V Y L D D A V K A A K F T K N H L V
721 ACTCACTACAACCAGGGTACATACATTGGGTCGGCCGTCGGATTGTATAAAGCGACCGGAAATGCCGTTTACCTCGATGACGCGGTCAAAGCCGCTAAATTCACCAAAAACCATCTGGTG
281 D S N G V L N Y E G P N G D L K G G K T I L M R N L A H L Q K T L D E T G Q Y P
841 GATTCAAACGGGGTGTTGAATTATGAAGGTCCCAACGGGGATCTGAAAGGCGGCAAAACGATCCTTATGCGCAATCTGGCCCATCTGCAAAAGACACTGGATGAAACCGGCCAGTACCCG
321 E F S A E F D E W L A F N I E M A W S H R N S D H I V D G N W A G Q L L S G T Y
961 GAATTCAGTGCTGAATTCGACGAATGGCTTGCATTCAATATCGAAATGGCCTGGAGTCATCGGAATTCGGATCATATCGTGGATGGAAACTGGGCGGGACAACTGCTGTCCGGAACCTAT
361 E S W S S A A A V Q A L N G I K P M E A E L H Y G V K N P F D K I E A E R Y N I
1081 GAATCCTGGTCATCGGCCGCAGCCGTTCAAGCTTTAAACGGTATCAAACCGATGGAAGCGGAGCTTCATTATGGTGTTAAAAACCCCTTCGATAAAATCGAAGCGGAACGCTACAATATC
401 G S G F V L E G A F E G S L Q L G G I Q H G S Y A A Y K N V D F G S D G A I G F
1201 GGGTCCGGTTTCGTTCTGGAAGGCGCGTTCGAAGGTTCGCTGCAATTAGGCGGAATACAGCATGGCTCTTATGCCGCTTACAAAAATGTGGACTTCGGATCCGACGGTGCGATCGGGTTC
441 I A R A S S G T G G G N I E I R L D S K D G P K V G T L N V E G T G D W N Q Y I
1321 ATTGCCAGAGCGTCCAGCGGAACAGGCGGAGGTAACATTGAGATCCGGCTTGATTCCAAGGATGGCCCCAAAGTCGGGACCTTGAACGTAGAGGGAACGGGCGACTGGAATCAATATATC
481 D A V T L L K D D Q G A P S T I T G V H D V Y L V F T K T N D D Y L F N L N W V
1441 GATGCCGTCACCCTCCTTAAAGATGACCAGGGAGCGCCGAGCACGATAACCGGCGTCCATGATGTGTATCTTGTCTTCACCAAGACGAACGACGATTATTTATTCAATTTGAACTGGGTT
521 K F T T T D P T E T D A Y A K L K A G N Y D S S E G L S K H A E F G Y L D A I H
1561 AAATTCACAACAACGGACCCGACGGAAACCGACGCCTATGCCAAGCTTAAGGCCGGAAATTACGACAGCAGCGAAGGACTTAGTAAACATGCCGAGTTCGGATATTTGGACGCAATCCAT
561 H N A Y A S Y E G I D F G S G A A G I T V H V A S G N Q G G T I E V K L D S L D
1681 CACAATGCCTATGCCTCTTATGAAGGAATTGATTTCGGATCTGGCGCGGCTGGTATCACGGTGCATGTGGCCAGCGGTAATCAAGGCGGCACGATCGAGGTTAAATTGGACAGTCTGGAT
601 G P T A G V I Q I P A L G S W D N W V D I M A N I D D T L A V G V H D V Y L V F
1801 GGACCTACCGCAGGGGTCATTCAAATTCCGGCGCTGGGAAGCTGGGATAACTGGGTCGATATCATGGCGAATATCGATGACACCTTAGCTGTAGGTGTTCACGACGTATACCTTGTTTTC
641 K G A N G S D Y P L N L E W F T F T T M K G K A R D A Y D K L E A E N Y T N G V
1921 AAGGGAGCGAACGGCAGCGACTACCCTCTCAATCTGGAGTGGTTCACCTTTACCACCATGAAAGGAAAAGCAAGGGATGCTTACGACAAGCTGGAAGCCGAAAACTATACAAACGGAGTA
681 G F G R E T G G G E T Y L A G M F G P N N P Y A M Y N Y I D F G S E S P T Q F H
2041 GGCTTCGGCAGAGAAACCGGCGGCGGGGAAACTTACCTGGCCGGGATGTTTGGACCGAATAACCCTTATGCTATGTACAATTACATCGATTTTGGCAGCGAAAGCCCTACCCAATTCCAT
721 V N A A S A T A G G T I E V R L D S L G G P V I A T A T V S G T G G W Q N F K V
2161 GTCAACGCAGCCAGCGCCACGGCTGGCGGGACAATCGAGGTCAGACTGGACAGTCTGGGCGGTCCGGTCATTGCAACAGCCACCGTCTCAGGAACCGGGGGCTGGCAAAATTTCAAAGTT
761 S S T D V T T P V T G K H I V F L S F K G G D W L Y N F D K F T F G D P A V F T
2281 TCCTCGACGGATGTAACGACCCCTGTAACGGGAAAACACATTGTATTCCTGTCTTTTAAAGGCGGAGACTGGTTATATAACTTTGATAAATTCACTTTTGGCGATCCGGCCGTATTCACA
801 E T P T P P M P E E D H V A P G E V E N V Q V K R G E G K M T L S W D G P Y D I
2401 GAGACTCCAACTCCTCCTATGCCTGAAGAGGACCATGTTGCTCCGGGCGAGGTAGAGAATGTTCAGGTGAAGCGCGGCGAGGGCAAGATGACGCTGTCTTGGGATGGTCCCTACGATATC
841 D A Q K V Q I T L R S N G Q Q V G D V I E V N R G I Q T A V I Q G I E A G K D Y
2521 GATGCTCAAAAAGTGCAAATTACGCTGCGGAGTAATGGACAGCAGGTCGGCGACGTCATTGAGGTAAATCGGGGAATTCAAACAGCGGTGATACAGGGAATCGAAGCGGGCAAGGACTAT
881 S L F I R S I D R S G N V S Q G V T I E V T D S P S F S L T V N G K S L E D G D
2641 TCGCTCTTCATCCGGTCCATTGATCGGTCCGGGAATGTATCGCAGGGAGTCACCATCGAGGTTACCGATTCTCCTTCCTTCTCTTTGACTGTTAATGGCAAGAGCTTGGAAGATGGCGAT
921 S L E D Y M A L S F K I M D T S A I Q L A E I T I G D K V Y T L D P L T K D A I
2761 TCTTTGGAGGATTACATGGCTCTCAGCTTCAAAATCATGGACACTTCTGCCATCCAATTAGCCGAAATAACCATCGGCGATAAAGTATATACGCTGGATCCATTGACCAAGGACGCTATC
961 D I D L A G N L G D I T A T V T T E D R S G N K T Q K T F Q F R V V T S V F S M
2881 GACATCGATTTGGCCGGGAATTTGGGCGACATAACGGCAACGGTTACGACTGAAGACCGCTCCGGTAACAAAACTCAGAAAACATTCCAGTTCCGGGTCGTGACCAGCGTGTTTTCCATG
1001 K Q L I D R F A D S G D V S G A L I P Q L T N A L N Q V Q H Q L D L E R V D H A
3001 AAACAATTAATCGACCGATTCGCGGATTCTGGGGATGTAAGCGGAGCGCTCATTCCTCAGCTGACCAACGCTCTTAACCAGGTACAGCATCAACTGGATCTGGAAAGAGTGGATCATGCC
1041 V K H M Q N F T K H L N K E A L G R N V S D Q A K T V L N T D A N S L L Q D W Q
3121 GTCAAGCATATGCAGAATTTCACCAAGCATCTGAATAAGGAGGCGTTGGGACGCAACGTCAGCGATCAAGCCAAAACGGTATTGAATACCGATGCGAACTCCCTGCTCCAAGATTGGCAG
1081 D G L E *
3241 GATGGCTTGGAGTAA

Figure S2

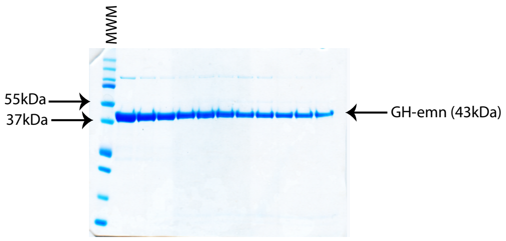

Figure S3

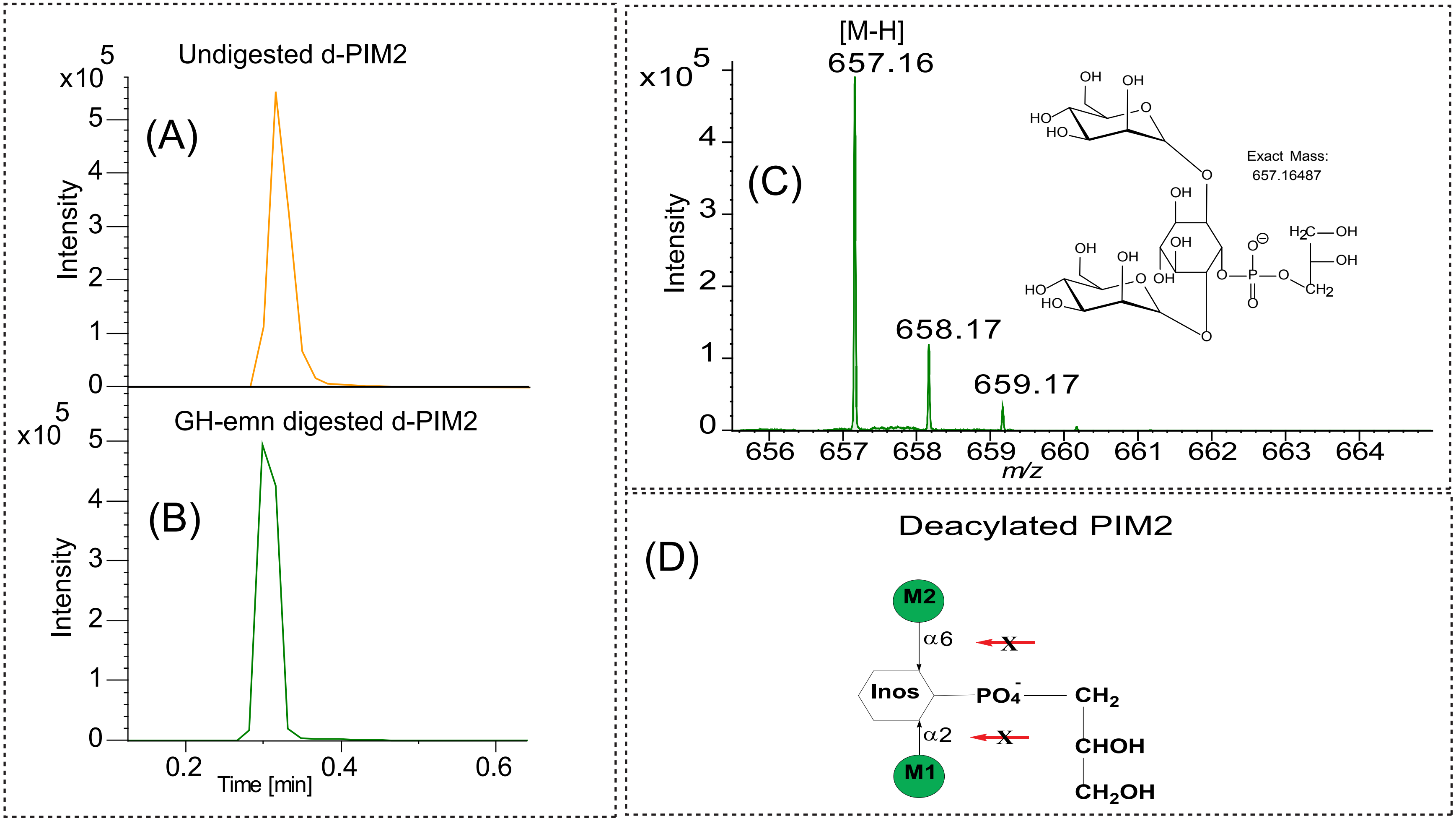

Undigested d-PIM2
(A)
x10^5
Intensity
GH-emn digested d-PIM2
(B)
Time [min]
(C)
[M-H]
657.16
658.17
659.17
m/z
Exact Mass:
657.16487
(D)
Deacylated PIM2
M2
α6
Inos
PO4-
CH2
α2
CHOH
M1
CH2OH

**Figure S3: LC/MS analysis of undigested and GM-emn-digested deacylated $PIM_2$.**

Extracted Ion Chromatogram of d-$PIM_2$ (*m/z* 657.16 [M–H]) before enzyme treatment (A) and after digestion with GH-emn (B). Panel (C) corresponds to the mass spectrum of d-$PIM_2$ after digestion with GH-emn. (D) Cartoon showing the lack of GH-emn cleavage site on d-$PIM_2$ (red arrows).
